# Diagnostic Tests for Alzheimer's Disease: Rationale, Methodology, and Challenges

**DOI:** 10.4061/2010/972685

**Published:** 2010-08-08

**Authors:** S. E. Mason, R. McShane, C. W. Ritchie

**Affiliations:** ^1^Centre of Mental Health, Imperial College London, London W6 8LN, UK; ^2^Nuffield Department of Medicine, University of Oxford, Oxford OX3 7LJ, UK

## Abstract

There has been a large increase in the amount of research seeking to define or diagnose Alzheimer's disease before patients develop dementia. If successful, this would principally have clinical benefits both in terms of treatment as well as risk modification. Moreover, a better method for diagnosing predementia disease would assist research which seeks to develop such treatments and risk modification strategies. The evidence-based definition of a diagnostic test's accuracy is fundamental to achieve the above goals and to address this, the Cochrane Collaboration has established a Diagnostic Test Accuracy group dedicated to examining the utility and accuracy of proposed tests in dementia and cognitive impairment. We present here the assumptions and observations underpinning the chosen methodology as well as the initial methodological approach decided upon.

## 1. Introduction

Acronyms were most useful when letters and reports were hand written or when one needed to save the ink on the typing ribbon. Nowadays, shorthand is unnecessary, especially when using it leads to confusion. Alzheimer's disease and Alzheimer's dementia are not synonymous, but when an author uses the acronym AD, it either suggests that the author believes (wrongly) that they are, or we (as the readers) are left in doubt as to which condition they were referring to.

Alzheimer's disease is a pathological diagnosis using a series of standardised criteria (e.g., Khachaturian, Consortium to Establish a Registry for Alzheimer's Disease and Reagan [1–3]) whereas Alzheimer's dementia is a clinical syndrome that is “possibly” or “probably” as a consequence of Alzheimer's disease [4]. Alzheimer's dementia is most commonly defined in a research setting using the NINCDS-ADRDA criteria which compared to results at autopsy, detects Alzheimer's disease with a sensitivity of 91–98% [5]. However, there are a group of clinical conditions probably mediated by Alzheimer's disease that do not satisfy the criteria for Alzheimer's dementia and these have been described as a premanifest or incipient Alzheimer's dementia. We prefer the term premanifest dementia and will use this term throughout. These conditions that will inevitably precede dementia are often indistinguishable from those caused by different pathologies and have therefore all been loosely referred to as Mild Cognitive Impairment; of which at least 16 prognostically weak definitions currently exist [6]. 

In Huntington's Disease research, the availability of an accurate diagnostic test has allowed investigation of the clinical correlates of the disease whilst the person is asymptomatic. Work of this nature has already, from baseline data alone, demonstrated neuroimaging and motor changes [7]. The “well” individuals who will later develop Huntingdon's disease are labelled “premanifest”, according to the TRACK-HD programme—a large collaborative European Research Programme which is investigating the earliest clinical and biomarker changes (plasma proteomics and neuroimaging) in this population. The overarching aim of their work is to be able to define means of identifying changes in the premanifest population that will act as surrogate markers of an intervention's efficacy at secondary prevention. Accordingly these interventions' efficacy can be measured reliably before the patients manifest overt signs of illness. In Alzheimer's dementia research we currently cannot describe the “premanifest” population (save for a small proportion of patients with fully penetrant, autosomal dominant mutations) and urgently need to be able to do so.

The main aims of the Cochrane Collaboration's Diagnostic Test Accuracy workstream within the Dementia and Cognitive Impairment Group are threefold. Firstly, to scrutinise the research of tests purporting to have diagnostic accuracy for Alzheimer's disease *in vivo*, secondly, using well rehearsed statistical techniques to derive a value for the test's diagnostic accuracy, and finally to highlight the deficiencies (where they exist) of extant research to improve the quality and direction of future primary research of diagnostic tests for Alzheimer's disease.

It should be made clear from the outset that the reviews can only reflect the primary research. Whilst the first few reviews are all of single tests or domains of tests (episodic memory, CSF and Plasma A*β* levels, Volumetric MRI, and ApoE status), the reviews will not be limited exclusively to single tests, be they clinical, genetic, or biomarker. We are aware that the optimal and only realistic way to make a diagnosis of premanifest dementia will be made by combining tests and such combination of tests (e.g., the CSF-Tau/A*β* ratio) can also be reviewed by authors registering an interest with the Cochrane Collaboration Dementia and Cognitive Impairment Group. These combinations can be evaluated using the DTA methodology described here. It is hoped also that by demonstrating the Diagnostic Accuracy of single tests through these reviews, this will help researchers to assemble test batteries and biomarker panels in the future from the strongest possible evidence base. Finally, the groups work is not limited to Alzheimer's disease, and we will welcome reviews on other forms of neurodegenerative disease both in their premanifest stages as well as a test or test batteries ability to distinguish between different types of dementia.

With that said, the following section describes the assumptions and observations that we have had to make and the methodology that will be enacted in an upcoming series of diagnostic test accuracy reviews by our group and its collaborators. This methodology describes the methodology for identifying Alzheimer's disease in patients before Alzheimer's dementia develops and is applicable to both single tests as well as scrutiny of combinations of tests.

## 2. Assumptions and Observations

### 2.1. Assumptions

#### 2.1.1. Assumption no. 1: Alzheimer's Dementia Does Not Develop Overnight

It is assumed that every patient who develops Alzheimer's dementia has to pass through a prodromal or incipient stage of mild cognitive impairment as described by various authors using several definitions [6]. The most common presentation for a patient with premanifest Alzheimer's dementia is a subjective episodic memory complaint, which is then diagnosed as mild cognitive impairment after an objective battery of neuropsychological tests demonstrate age-matched deficiencies. However as Matthews et al. described, these definitions hold little prognostic significance with considerable variations in the level of impairment and outcome at two years [6]. Most definitions of mild cognitive impairment are cross sectional which over time may develop along one of five clinical courses. Though these courses are not necessarily specific to any one disease or illness trajectory. For example, depression or other psychiatric illness may exhibit courses 3, 4, and 5 below.

1. Continued deterioration in cognition and functional decline to satisfy criteria for Alzheimer's dementia.
2. Conversion to another subtype of dementia, for example, Lewy body or vascular dementia.
3. Continued deterioration in cognition which does not go on to satisfy criteria for a dementia.
4. No conversion, but stability of deficit with no recovery or progression.
5. Recovery of cognitive abilities.

#### 2.1.2. Assumption no. 2: Risk Factor Modification Will Work Best Early in the Disease Course

There has been high quality evidence produced of late that suggests modification of identified epidemiological risk factors for dementia (e.g., decreased oestrogen levels in the menopause [10, 11]) have little benefit and indeed may have safety concerns as treatments once dementia has developed [12]. This and other similar observations have led to the belief that there may well be a critical window, earlier in the course of the underlying disease, for intervention or risk factor modification which may include optimising cardiovascular health, increasing exercise, minimising the risk of depression, improving diet, increasing intellectual activity and reducing the risks for diabetes and stroke [13].

#### 2.1.3. Assumption no. 3: Patients Asking for Help Want to Know What Is Causing Their Symptoms

People who have capacity and knowledge that they have premanifest Alzheimer's dementia will be in a position to create lasting powers of attorneys, advanced directives and engage in financial, social and lifestyle planning whilst well. Therefore, it is further assumed that there is merit for the patient in identifying Alzheimer's disease prior to dementia developing. They also have the opportunity to make advanced decisions regarding engaging in clinical research.

#### 2.1.4. Assumption no. 4: Disease Modifying Drugs Will Probably Work Best in Early Disease

Common sense dictates the earlier one starts a course of treatment for any disease the better, though being mindful of any side effects that may outweigh the initial benefits. No potentially disease modifying therapies for Alzheimer's disease have demonstrated clear efficacy in late stage clinical trials in patients with Alzheimer's dementia, though several still retain promise [14]. Whilst this may be due to the drugs simply not working, the targets being wrong or the tools to measure effect being insensitive to change; it is also possible that these drugs will only work when the disease process is less advanced. This concept has been described in multiple sclerosis, where disease modifying therapies are ineffective in patients whose disability has progressed to a more severe level [15]. In essence, testing this hypothesis will require recruiting a population who definitely have early stage disease to be able to develop disease modifying drugs and thereafter use this population as the target for the clinical intervention. Such intervention may lead to the secondary prevention of Alzheimer's dementia by manipulating the progression of the predementia pathology; with great human, social, and economic benefit.

## 3. Observations

### 3.1. Observation no. 1: The Gold Standard for Diagnosis

The development of a new diagnostic test for any condition is predicated on the ability to determine the accuracy of the new or index test against the gold or reference standard. This creates problems specific to Alzheimer's disease in that there is no *in vivo* gold standard, currently its premortem presence is “probable” or “possible” based on an array of symptoms described as Alzheimer's dementia and neuroimaging findings. It is worth considering that the applicability and acceptability of clinical criteria such as NINCDS-ADRDA are partly based on their concordance with pathological studies. Brain biopsy (the means to achieving the gold standard) is not ethical for this purpose unlike other invasive gold standard tests (e.g., liver biopsy to determine the accuracy of fibroscan for cirrhosis). As the aim of the Cochrane reviews are to accurately diagnosis disease before dementia develops, the fact that a patient develops Alzheimer's dementia (NINCDS-ADRA criteria) is taken as *ipso facto *proof that at the point of testing, disease was present. We recognise that an alternative reference standard is needed to be used and this choice was subject to much debate and discussion by the Cochrane Dementia and Cognitive Impairment Editors. We hope that over time a better reference or gold standard will emerge, until such time though and with inherent limitations, the conversion to NINCDS-ADRDA Alzheimer's dementia will be used as the gold standard.

### 3.2. Observation no. 2: The Gold Standard for Alzheimer's Disease

Accepting the assumption that there is a group of patients at the predementia stage of Alzheimer's disease manifesting cognitive impairment and that there are currently no means to affect the trajectory of their disease then conventionally, it is assumed that the development of Alzheimer's dementia represents the necessary level of proof that at the pre-dementia stage they had Alzheimer's disease. If this is accepted, then this overcomes the problem of the index test and reference test being separated over time. Again, although not ideal, it is at this stage considered the optimal solution to the definition of a gold standard. Accordingly, the reference standard is the development of clinically diagnosed Alzheimer's dementia or other dementias depending upon the aims of specific reviews. This, however, does create problems considering whether the test is actually diagnostic or prognostic. If it is the latter, then we are asking whether the test at baseline predicts a pathway of decline, stability or improvement. However in the context of the Cochrane Diagnostic Test Accuracy reviews we are only interested in the ability of the test to identify underlying Alzheimer's disease and therefore be associated with the development of Alzheimer's dementia. It has no bearing what the prognosis (clinical, behavioural or functional) of the Alzheimer's disease is for any given individual who returns a positive test but rather whether or not they have Alzheimer's disease in their brain confirmed by the test in question.

## 4. The Diagnostic Test Accuracy Process—What Is Being Proposed as a Methodology?

There are three stages in developing clinically applicable diagnostic tests or criteria for premanifest Alzheimer's dementia (Table 1).

1. The testing of single or individual diagnostic tests which may be laboratory based, clinical, genetic or radiological in nature.
2. Applying diagnostic test accuracy statistical enquiry on a battery or collection of the above tests (e.g., Devanand et al. [16]) akin to the development of the antenatal triple test for Down's Syndrome.
3. Consensus statement by clinical specialists using what the assembled and invited experts deem to be the most promising imaging, neuropsychological and laboratory tests. For example those described by Tabert et al. and Dubois et al. [8, 9].

The remainder of this paper will focus on the efforts of the Cochrane Collaboration to determine the accuracy of a single diagnostic test. It is recognised that it is highly unlikely that one test for a complex disorder will have sufficient diagnostic accuracy however, each test will be a constituent of a battery that may be developed from the strongest candidates be they biomarker, genetic, or clinical. Over time the same methodology will be applied to test batteries as and when they arise. As the Cochrane Collaboration is widely known for its reviews of randomised controlled trials (which helped in the drive towards the CONSORT criteria for the reporting of clinical trials) the Diagnostic Test Accuracy review will have the following quality features as set out in the Cochrane Collaborations DTA Handbook [17].

1. Careful delineation of the diagnostic question.
2. Systematic searching.
3. Making comparisons between tests concerning their global accuracy.
4. Estimating the accuracy of a test operating at a particular threshold.
5. Understanding why the results of studies vary.

The purpose of a Cochrane Diagnostic Test Accuracy review is to use meta-analysis techniques to establish a confidence around test accuracy which informs diagnosis of the condition in question. This is done by using a meticulous, objective search strategy in order to identify data from high quality studies which can be combined in meta-analysis. 

The principle of this Diagnostic Test Accuracy review methodology is to look for studies which document the test of interest in patients with any of the definitions of Mild Cognitive Impairment and then compare the results of the test between those who convert to Alzheimer's dementia and those with different outcomes, in order to determine its diagnostic accuracy when applied at the Mild Cognitive Impairment stage. The undertaking of a successful Diagnostic Test Accuracy review requires a clear description of the studies suitable for inclusion before the search, to keep bias to a minimum and to ensure only the studies of highest quality are analysed.

In order to achieve the objectives of a DTA review, three major points have to be defined.

1. The Test to Be Assessed
  - This can include plasma, urine or cerebrospinal fluid biomarkers and genetic, radiological and well defined neuropsychological assessments. As well as combinations of these.
2. The Population to Study
  - We concluded that the tests should have been applied in any of the referenced definitions of cognitive impairment believed to be due to neurodegeneration [6]. Only by including all such definitions can we define the tests ability to identify Alzheimer's disease from the absence of neurodegenerative disease, but also its ability to identify which type of neurodegenerative disease is present.
3. The Condition under Interest
  - It is a dilemma that there is no “gold standard” ante mortem diagnostic test for Alzheimer's disease. Post-mortem provides an apparently definitive diagnosis however in the extant literature, few baseline biomarker levels are recorded in those individuals who then have a post mortem. For future studies, the reference standard will be conversion to Alzheimer's dementia based on the NINCDS-ADRDA criteria, which is “probably” or “possibly” a result of Alzheimer's disease. This of course implies a restricted view of the outcome to one particular form of dementia. It could be argued that a broader concept of dementia would be acceptable as this is what is (in effect) most relevant to the patient, their carer and their doctor. This is acceptable as long as each review explicitly states the final condition of interest. The Cochrane Collaboration Dementia and Cognitive Impairment DTA Group is not only interested in DTA's for Alzheimer's disease but also reviews for all other dementias and neurodegenerative disease associated with cognitive impairment.

A sensitive search strategy is then employed to form the basis of the systematic review. After independent assessment for inclusion in the DTA review, the results are analysed. The analysis of DTA reviews is more challenging than with intervention reviews due to the binary classifications of sensitivity and specificity and the tradeoff between the two measures. The statistical method behind combining the results is described in each review and the data from the two-by-two tables will be used to calculate the sensitivity, specificity, positive and negative likelihood ratios, and diagnostic odds ratio for each study. Individual study results are presented graphically by plotting estimates of sensitivities and specificities in Receiver Operating Characteristic (ROC) space. If more than one threshold is reported by the study authors, then the two-by-two table for one threshold will be chosen to incorporate in the meta-analysis.

## 5. Summary of Difficulties and Dilemmas with Diagnostic Test Accuracy Reviews in Alzheimer's Disease

The main difficulties to be faced are threefold

1. The use of clinical criteria occurring after the test as a gold standard.
2. The reliability of the criteria for participant inclusion in studies.
3. The relevance of the test of interest and the defined population.

The most accurate reference standard would be to use Alzheimer's disease diagnosed by brain biopsy in order to combat the current lack of an ante-mortem “gold standard” test. However, this is currently unethical in the live patient purely for the purpose of research. Also important are the multiple definitions for the premanifest stage of Alzheimer's dementia. The criteria to diagnose MCI vary and are not uniformly applied, resulting in a dissimilar predictive power for progression to Alzheimer's dementia between definitions [6]. In other words, each definition of a clinical state—when defined gives a different pretest probability for Alzheimer's disease. This will lead to heterogeneity in the review which will be documented and explained where possible. 

An accurate diagnostic test for Alzheimer's disease in the patient with premanifest Alzheimer's dementia may be imminent. However based on the methodology of the Diagnostic Test Accuracy review this will only be demonstrable in a defined population with diagnosed cognitive impairment. There is an argument that those with no symptoms what-so-ever (truly premanifest) should be targeted because the benefit of potential treatments or risk modification will be augmented. This will hopefully be the next step if accurate diagnosis of disease can be achieved in the patients already exhibiting and complaining of cognitive impairment.

## 6. Conclusions

The objective of this paper is to ensure that in future years the Cochrane Collaboration will have reviewed and constructed a list of accurate diagnostic tests and test batteries, such that clinicians will be able to accurately identify and intervene with best practice in impaired patients with an earlier disease stage prior to the development of disabling dementia. Secondly, researchers will have a means to define a premanifest Alzheimer's disease population to direct disease modifying therapies towards and, finally, we hope that in being critical of current diagnostic test methodologies we will have some impact on generally improving the quality of primary research of diagnostic tests in dementia over time. 

We believe that there are many approaches and dilemmas to consider, which can only be overcome by using dispassionate, unbiased, stringent, and evidence-based methods. Using these methods (which may evolve over time), we can optimise the chances of achieving accurate diagnosis of Alzheimer's disease at an earlier stage than is currently possible when the patients and their carers can derive maximal benefit from future treatments, psychosocial interventions, as well as lifestyle changes and risk modification.

## Figures and Tables

**Table 1:** Advantages and disadvantages of different approaches to developing an accurate diagnostic test.

	Advantages	Disadvantages
Single test	Fast and often easy to perform. Acceptable to the patient Often inexpensive Reproducible and quantitative	The Diagnostic Odds Ratio (DOR) may not be clinically significant with only one test in such a complex disorder Results may only be significant in a highly defined population where pre-test probability is already high
Multiple tests	More tests should each add incremental benefit to the constituent single tests and increase DOR accordingly Can combine tests from different domains, for example genetic, clinical, radiological, and proteomic	Patient must undergo many tests to complete the battery Much research is required before these batteries are available May be expensive when including specific assays and imaging Results may only be significant in a defined population where pretest probability already high
Consensus	Such criteria are easy to produce as do not rely on any systematic, unbiased and comprehensive literature review or testing. Such criteria have already been described [8, 9]	While these criteria are produced and highlight the need for validation, they are often accepted as being valid before this process is undertaken or completed They tend to represent a series of well educated, best guesses. Diagnostic criteria then need testing but application of consensus criteria tends to be piecemeal due to multiple “either/or” criteria. Lack the rigour of well defined tests with thresholds and inherent objectivity Most require many tests and time from clinical and radiological specialists which may make it hard to reproduce between centres Resources required are expensive. The need for further verification of current criteria have been highlighted [8, 9]
